# Supplementary material for: Novel Antibiofilm Inhibitor Ginkgetin as an Antibacterial Synergist against Escherichia coli
Source: Int J Mol Sci. 2022 Aug 8;23(15):8809. doi: 10.3390/ijms23158809 (PMC9369100; doi:10.3390/ijms23158809)
Supplement: Supplementary file 1 [file ijms-23-08809-s001.zip › ijms-1859534-supplementary.pdf]

Novel Antibiofilm Inhibitor Ginkgetin as an Antibacterial Synergist against *Escherichia coli*

Yubin Bai[1,2,3], Weiwei Wang[1,2,3], Mengyan Shi[1,2,3], Xiaojuan Wei[1,2,3], Xuzheng Zhou[1,2,3], Bing Li[1,2,3], Jiyu Zhang[1,2,3*]

1 Key Laboratory of New Animal Drug Project of Gansu Province, Lanzhou 730050, China; 82101191226@caas.cn (Y.B.); wangweiwei@caas.cn (W.W.); 82101202383@caas.cn (M.S.); weixiaojuan@caas.cn (X.W.); zhouxuzheng@caas.cn (X.Z.); libing@caas.cn (B.L.)
2 Key Laboratory of Veterinary Pharmaceutical Development, Ministry of Agriculture, Lanzhou 730050, China
3 Lanzhou Institute of Husbandry and Pharmaceutical Sciences, Chinese Academy of Agricultural Sciences, Lanzhou 730050, China
* Correspondence: zhangjiyu@caas.cn

Table S1. MIC of Antibiotics Colistin B, Colistin E, and Gentamicin to different *E. coli* Species

antibiotics	MIC(μg/mL)					
	ATCC 25922	C83654	XJ24	O149	KD-13-1	O101
Gentamicin	1	2	0.5	16	1	32
Colistin B	0.5	512	4	256	16	4
Colistin E	0.5	1024	4	512	4	2

Table S2. The primer sequences used in qRT-PCR

Genes	sequence (5′ to 3′)	Product size (bp)
gapA-F	CCAGGACATCGTTTCCAAC	103
gapA-R	GGTGGTCATCAGACCTTCG	103
csgA-F	CAGATGTTGGTCAGGGCTCAGATG	127
csgA-R	CCGCCACCGAATTGTTTAACTGTC	127
csgD-F	TGATGAACAACGAACGAGCGATCTC	146
csgD-R	GCTTGCCAGTTACCTGATTACACATTC	146
flhC-F	ATGCTGCCATTCTCAACCGACTG	117
flhC-R	CGCATCGACGCCATTACACAAAC	117
flhD-F	CGTTAGCGGCACTGACTCTTCC	107
flhD-R	TTGCGTCAACTGAGTAATCGTCTGG	107
fliC-F	TTACCAACCTGAACAACACCACTACC	90
fliC-R	ACATATTGGACACTTCGGTCGCATAG	90
fliM-F	CCGACCAACCTGAACCTTATCCATC	88
fliM-R	CCACGGCGATAAACACCAGACTC	88
luxS-F	GAAAACAATGAACACCCCGCATGG	92
luxS-R	TCCCTCTTTCTGGCATCACTTCTTTG	92
lsrB-F	AGTGCTGACCTGGGACTCTGATAC	99
lsrB-R	GCCATATCCACCAACATACCTCCTAAC	99
lsrK-F	GATGAACCTACCGCCTCGCTTAC	90
lsrK-R	AACAATACCCACGCCAGTAGCAAG	90
lsrR-F	ACCACAACAGATGCTGGCGATTG	143
lsrR-R	GCTGCCCGATTCCCGTCATATAAG	143
